# Adverse pregnancy outcomes associated with first‐trimester exposure to angiotensin‐converting enzyme inhibitors or angiotensin II receptor blockers: A systematic review and meta‐analysis

**DOI:** 10.1002/prp2.644

**Published:** 2020-08-19

**Authors:** Nida Buawangpong, Supanimit Teekachunhatean, Nut Koonrungsesomboon

**Affiliations:** ^1^ Department of Family Medicine Faculty of Medicine Chiang Mai University Chiang Mai Thailand; ^2^ Department of Pharmacology Faculty of Medicine Chiang Mai University Chiang Mai Thailand; ^3^ Musculoskeletal Science and Translational Research Center Chiang Mai University Chiang Mai Thailand

**Keywords:** adverse pregnancy outcome, angiotensin II receptor blocker, angiotensin‐converting enzyme inhibitor, congenital malformation

## Abstract

This study aimed to determine the effects of prenatal exposure to angiotensin‐converting enzyme inhibitors (ACEIs) or angiotensin II receptor blockers (ARBs), particularly when exposure is limited to the first trimester of pregnancy, on adverse maternal and neonatal outcomes. A systematic search was performed on four databases, that is, PubMed, Scopus, Web of Science, and Cochrane Library, to identify relevant articles published up to December 31, 2019. Included studies were limited to original investigations assessing the association between prenatal exposure to ACEIs/ARBs and adverse pregnancy outcomes. Odds ratios were used as a summary effect measure. Pooled‐effect estimates of each outcome were calculated by the random‐effects meta‐analysis. The main outcomes included overall and specific congenital malformations, low birth weight, miscarriage, elective termination of pregnancy, stillbirth, and preterm delivery. Of 19 included articles involving a total of 4 163 753 pregnant women, 13 studies reported an increased risk of, at least, one adverse pregnancy outcome in pregnant women who were exposed to ACEIs/ARBs. Meta‐analysis revealed a significant association between overall congenital malformations and first trimester‐only exposure to ACEIs/ARBs (OR = 1.94, 95% CI = 1.71‐2.21, *P* < .0001). Cardiovascular malformations, miscarriage, and stillbirth also provided a significant relation with ACEI/ARB exposure. In conclusion, prenatal exposure to ACEIs/ARBs in the first trimester of pregnancy was found to be associated with an increased risk of adverse pregnancy outcomes. Women of reproductive age should be aware of the potential teratogenic risks of these drugs if they become pregnant.

**Abbreviations**
ACEI: angiotensin‐converting enzyme inhibitor
ACR: assumed comparator risk
ARB: angiotensin II receptor blocker
CI: confidence interval
CNS: central nervous system
CVS: cardiovascular system
ETOP: elective termination of pregnancy
FDA: Food and Drug Administration
GRACE: good research for comparative effectiveness
LBW: low birth weight
OAH: other antihypertensive medications
OR: odds ratio
PRISMA: preferred reporting items for systematic reviews and meta‐analyses
RAAS: renin‐angiotensin‐aldosterone system
RevMan: review manager
RoB 2: a revised tool for assessing risk of bias in randomized trials
RR: risk ratio

## 1 INTRODUCTION

Angiotensin‐converting enzyme inhibitors (ACEIs) and angiotensin II receptor blockers (ARBs), an alternative for ACEI‐intolerant patients, are commonly used for the treatment of cardiovascular disease whereby the renin‐angiotensin‐aldosterone system (RAAS) is involved in its pathophysiology.^1^, ^2^ ACEIs/ARBs modulate the RAAS by either inhibiting an enzyme responsible for the conversion of angiotensin I to angiotensin II or by antagonizing the effects of angiotensin II at its receptors. As such, ACEIs/ARBs are beneficial to enhanced natriuresis, reduced afterload, and deferral of cardiovascular remodeling, making them useful for various cardiovascular conditions, such as hypertension, heart failure, and postmyocardial infarction.^3^, ^4^, ^5^ ACEIs/ARBs are, thus, one of the most widely prescribed drug classes, with hundreds of thousands of patients worldwide who are exposed to each year.^6^, ^7^

In 1980s‐1990s, there were a series of cases reported to the US Food and Drug Administration (FDA) indicating that ACEIs/ARBs are teratogens when being used in the second and third trimesters of pregnancy.^8^ Evidence suggests a relationship between neonatal adverse outcomes (ie, oligohydramnios and other adverse outcomes secondary to impaired fetal kidney development) and ACEI/ARB exposure.^9^, ^10^ A “black box” warning issued by the US FDA in 1992 has raised awareness of the teratogenic potential of ACEIs/ARBs, and the second and third trimesters of pregnancy are considered a contraindication to the use of ACEIs/ARBs accordingly.^11^ In the 2013 Report of the American College of Obstetricians and Gynecologists’ Task Force on Hypertension in Pregnancy, it has been recommended not to use ACEIs/ARBs in women of reproductive age if there is no compelling reason.^12^ Despite such a feature in the labeling of the potentially teratogenic medications, several cases of fetal exposure to ACEIs/ARBs have been reported thereafter.^13^, ^14^, ^15^ ACEI/ARB exposure during pregnancy is still highly prevalent in many settings.^16^, ^17^

Up to the present time, it still remains unclear whether ACEIs/ARBs are teratogenic if exposure to these drugs is only limited to the first trimester of pregnancy.^18^, ^19^, ^20^ Several epidemiologic studies report inconsistent results on the teratogenic effects of first‐trimester ACEI/ARB exposure in humans.^17^, ^21^, ^22^ Given the increasing incidence of hypertension and conditions in which ACEIs/ARBs are often indicated, systematic investigations on the potential teratogenic consequences of ACEI/ARB exposure during early pregnancy are highly needed to provide more concrete guidance for the use of ACEIs/ARBs in women of reproductive age.^23^, ^24^ Should ACEI/ARB exposure during the first trimester of pregnancy be considered nonteratogenic, female patients of childbearing potential could be safely prescribed either an ACEI or an ARB, provided that they are advised of the risks involved and can switch the drug to other alternatives within a few weeks after conception. On the other hand, if exposure to ACEIs/ARBs during the first trimester of pregnancy is associated with an increased risk of congenital malformations or adverse maternal outcomes, the use of ACEIs/ARBs in women of reproductive age should be discouraged, particularly given the availability of alternative medications to treat their conditions.^25^

The objective of the present study was to determine the effects of prenatal exposure to ACEIs/ARBs, particularly when exposure is limited to the first trimester of pregnancy, on adverse maternal and neonatal birth outcomes by means of systematic review and meta‐analysis.

## 2 METHODS

This study conformed to the preferred reporting items for systematic reviews and meta‐analyses (PRISMA) guidelines.^26^ The study protocol was prospectively registered at the PROSPERO international prospective register of systemic reviews in health and social care (CRD42019140107).

### 2.1 Search strategy and eligibility criteria

Initial literature searches were systematically performed in four major search engines, that is, PubMed, Scopus, Web of Science, and Cochrane Library, in September 2019, and a repeated search was updated on December 31, 2019. The terms related to adverse pregnancy outcomes (including congenital malformations, teratogens, fetus, and pregnancy) and ACEIs/ARBs (including all generic drug names based on Micromedex) were used to develop a comprehensive search strategy, with no language restriction, to identify all relevant articles. A number of medical subject headings were combined using the ‘OR’ operator; the results of the two searches (ie, adverse pregnancy outcomes and ACEIs/ARBs) were combined with the ‘AND’ operator. The reference lists of selected articles were screened manually in search of additional articles, if any.

Relevant studies were selected based on the following criteria: (a) a study involved pregnant women; (b) there was ACEI/ARB exposure during pregnancy; and (c) either adverse maternal outcomes or neonatal birth outcomes, or both, were reported. Included studies were limited to original investigation performed on humans. No restriction was made with respect to study design or subjects’ underlying conditions. Studies lacking a control group (eg, case reports, case series, or expert opinion) and review articles (including systematic reviews) were excluded. All studies deemed suitable were retrieved and reviewed independently by two authors to determine study eligibility. Study selection was carried out by two authors independently; disagreements were resolved through discussion and consensus.

### 2.2 Data extraction and quality assessment

Two authors independently extracted data from original full‐text articles using a standardized data collection form. The data extracted included (a) first author, (b) publication year, (c) study design, (d) study setting/location, (e) study period, (f) stage of pregnancy, (g) number of participants, (h) exposure (ie, ACEIs or ARBs), (i) control (ie, exposure to other antihypertensive drugs or nonexposure), and (j) outcome of interest (ie, overall and specific congenital malformations, low birth weight (LBW) (birth weight < 2500 g), miscarriage or spontaneous abortion, elective termination of pregnancy (ETOP), stillbirth, and preterm delivery). In cases where data were missing in an original publication or required clarification, attempts were made by e‐mail contact with the corresponding author.

The quality of included studies was evaluated using the standardized Good Research for Comparative Effectiveness (GRACE) checklist for observational studies and a revised tool for assessing the risk of bias in randomized trials (RoB 2) for randomized‐controlled trials.^27^, ^28^

### 2.3 Data analyses

The exposure of interest was maternal exposure to ACEIs/ARBs during any trimesters of pregnancy or during the first trimester only, and the outcome of interest was adverse pregnancy outcomes, including both maternal and neonatal outcomes. The first trimester‐only exposure was defined as any use of ACEIs/ARBs from the last menstrual period to the third month of pregnancy. An exposure cohort was defined as a group of pregnant women who were exposed to ACEIs/ARBs (ACEI/ARB group), while a control cohort was those who were exposed to other antihypertensive medications (OAH group) or those with no exposure to any antihypertensive drugs (nonexposure group). Extracted relevant data were tabulated in a 2 × 2 contingency table. Odds ratios (ORs) were used as a summary measure for meta‐analysis of dichotomous outcomes. Risk ratios (RRs) were calculated from ORs using the following formula for ease of interpretation: RR = OR/ [1 – ACR × (1 – OR)]; given that the assumed comparator risk (ACR) is the risk that the outcome of interest occurred in the control group.

Pooled‐effect estimates of each outcome of interest were calculated by the Mantel‐Haenszel random‐effects meta‐analysis. A cumulative meta‐analysis was conducted to determine whether each study added to the pool affected the overall estimate changes. Statistical heterogeneity among included studies was assessed using the Cochran's *Q* test and the percentage of total variability across studies due to heterogeneity (*I* ^2^ value). Subgroup analyses were conducted to determine the impact of ACEI/ARB exposure on adverse maternal and neonatal birth outcomes when exposure was limited to the first trimester of pregnancy.

Potential bias from small‐study effects (eg, publication bias) was assessed through visual examination of funnel plots displaying the log OR of individual studies on the horizontal axis and its standard error on the vertical axis.^29^ A rank correlation test and a linear regression test were applied to identify any potential publication bias in a meta‐analysis with 10 or more included studies.^30^, ^31^ Sensitivity analyses on the impact of study design, drug classes, and exclusion of a single study from meta‐analysis as well as the impact of fixed‐effect or random‐effects models on summary measures were performed.

All tests were two‐tailed; *P* < .05 was considered statistically significant. Quantitative syntheses of the data were done in Review Manager (RevMan) version 5.3. Cumulative meta‐analyses were performed in chronological order using a standard software package (Stata, version 16.0; StataCorp). Formal tests for funnel plot asymmetry were performed using the jamovi project (2019), jamovi version 1.0 (Computer Software), retrieved from https://www.jamovi.org.

## 3 RESULTS

**FIGURE 1:**
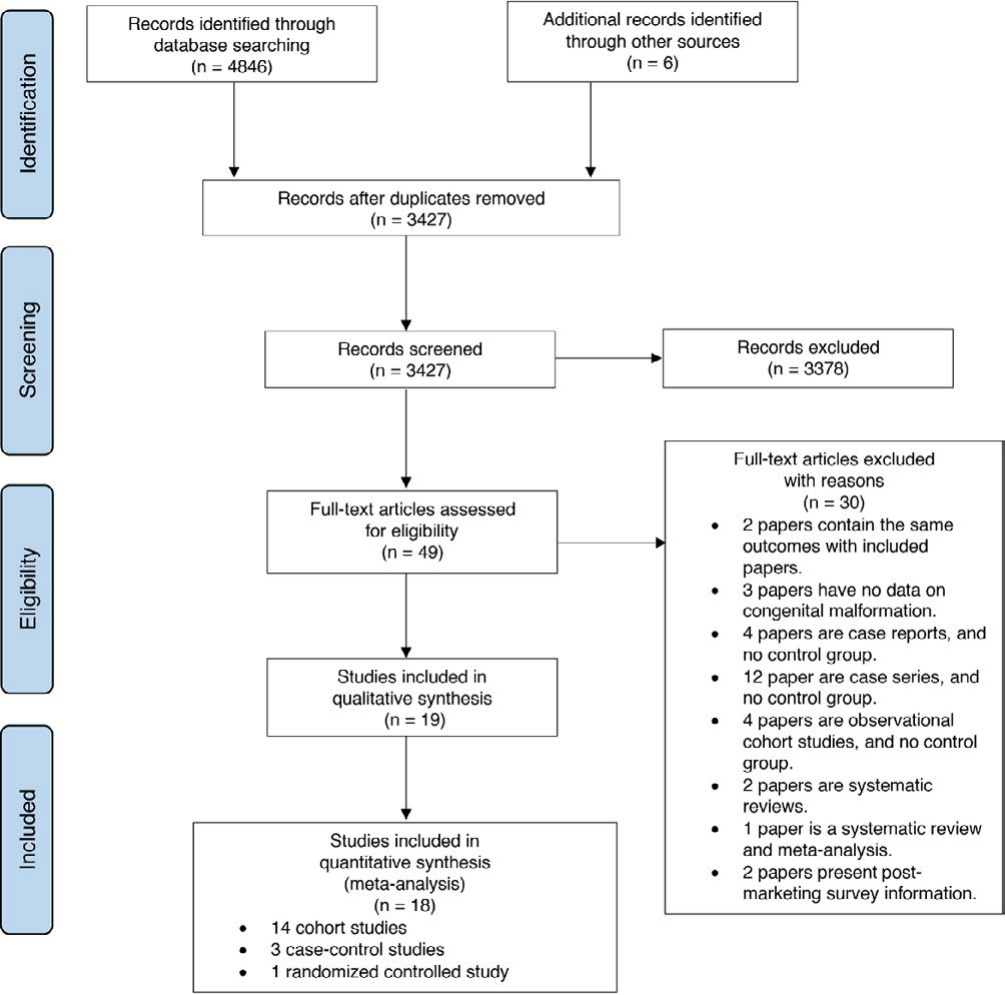
Flow diagram

Of 3427 potentially relevant records identified through the systematic search, 49 full‐text articles were retrieved and examined for eligibility. A total of 19 articles, published between 1992 and 2018, were included for data extraction, with 18 articles that enabled quantitative analysis (Figure 1). Characteristics of 19 studies are presented in Table 1, ^32^, ^33^, ^34^, ^35^, ^36^, ^37^, ^38^, ^39^, ^40^, ^41^, ^42^, ^43^, ^44^, ^45^, ^46^, ^47^, ^48^, ^49^, ^50^: 15 are observational cohort studies, three are case‐control studies, and one is a randomized‐controlled trial, all of which were classified as ‘sufficient quality’ or ‘low risk of bias’ studies (Table S1). Relevant studies were conducted in North America (n = 9), Europe (n = 9), or Australia (n = 2). Data syntheses involved a total of 4 163 753 pregnant women, with 7075 exposed to ACEIs/ARBs, 25 379 to other antihypertensive drugs, and 3 782 450 nonexposed individuals. Around two thirds of studies included in qualitative analysis (13/19) reported an increased risk of, at least, one adverse pregnancy outcome of interest in pregnant women with ACEI/ARB exposure (Table S2).

**TABLE 1:** Study characteristics

Study	Year	Study design	Stage of pregnancy	Locations	Periods	Exposure	Comparator
Ahmed et al^32^	2018	Retrospective cohort	First trimester	Australia	2005‐2012	ACEIs/ARBs	Methyldopa
Banhidy et al^33^	2011	Case‐control	Any trimesters	Hungary	1980‐1996	Captopril	OAH; Nonexposure
Bateman et al^34^	2017	Retrospective cohort	First trimester	United States	2000‐2010	ACEIs	Nonexposure
Caton et al^35^	2009	Case‐control	First trimester	United States	1997‐2003	ACEIs/ARBs	OAH; Nonexposure
Chintamaneni et al^36^	2018	Retrospective cohort	Any trimesters	United States	2003‐2014	ACEIs (mostly Lisinopril)	Nonexposure
Colvin et al^37^	2014	Retrospective cohort	Any trimesters	Australia	2002‐2005	ACEIs	Nonexposure
Cooper et al^38^	2006	Retrospective cohort	First trimester	United States	1985‐2000	ACEIs	OAH; Nonexposure
Cournot et al^39^	2006	Prospective cohort	First trimester	France	n/a	ACEIs	Nonexposure
Diav‐Citrin et al^40^	2011	Prospective cohort	First trimester	Israel; Italy	1994‐2007; 1990‐2008	ACEIs	OAH; Nonexposure
Fisher et al^41^	2017	Case‐control	First trimester	United States	1997‐2011	ACEIs/ARBs	OAH; Nonexposure
Hoeltzenbein et al^42^	2018a	Prospective cohort	First trimester^1^	Germany	2000‐2014	ACEIs	Methyldopa; Nonexposure
Hoeltzenbein et al ^43^	2018b	Prospective cohort	First trimester^1^	Germany	2000‐2014	ARBs	Methyldopa; Nonexposure
Lennestal et al^44^	2009	Retrospective cohort	First trimester	Sweden	1995‐2006	ACEIs/ARBs	OAH; Nonexposure
Li et al^45^	2011	Retrospective cohort	All trimesters; First trimester; Second or third trimester	United States	1995‐2008	ACEIs	OAH; Nonexposure
Malm et al^46^	2008	Retrospective cohort	First trimester	Finland	1996‐2001	ACEIs	OAH; Nonexposure
Moretti et al^47^	2012	Prospective cohort	First trimester	Canada	n/a	ACEIs/ARBs	OAH; Nonexposure
Piper et al^48^	1992	Retrospective cohort	All trimesters	United States	1983‐1988	ACEIs	n/a
Porta et al^49^	2011	Randomized‐control^2^	First trimester	Italy, USA, UK, Denmark, Sweden	2001‐2008	Candesartan	Nonexposure
Vasilakis‐Scaramozza et al^50^	2013	Retrospective cohort	First trimester	United Kingdom	1991‐2002	ACEIs	OAH; Nonexposure

Abbreviations: ACEIs, angiotensin‐converting enzyme inhibitors; ARBs, angiotensin II receptor blockers; n.a., not available; OAH, other antihypertensive medications.

^1^ No longer than gestational week 20.

^2^ Data derived from three randomized, placebo‐controlled trials (ie, DIRECT‐Prevent 1, DIRECT‐Protect 1, and DIRECT‐Protect 2).

**FIGURE 2:**
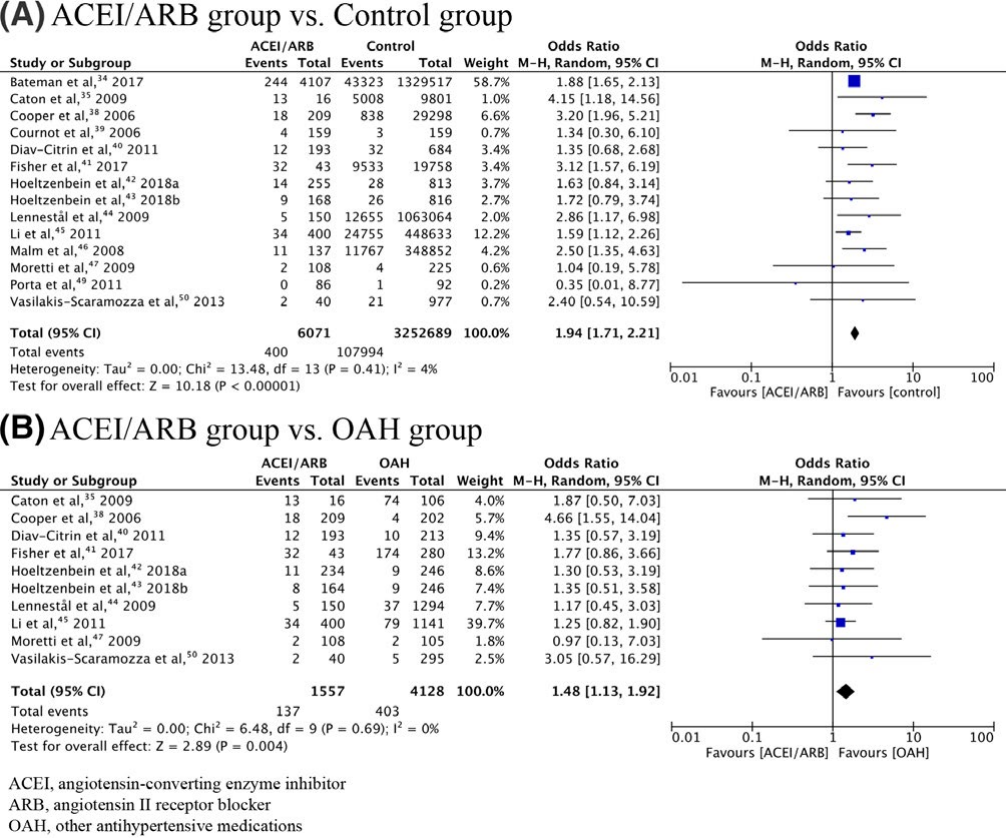
Forrest plot of overall congenital malformations in first trimester‐only exposure to ACEI/ARB

**FIGURE 3:**
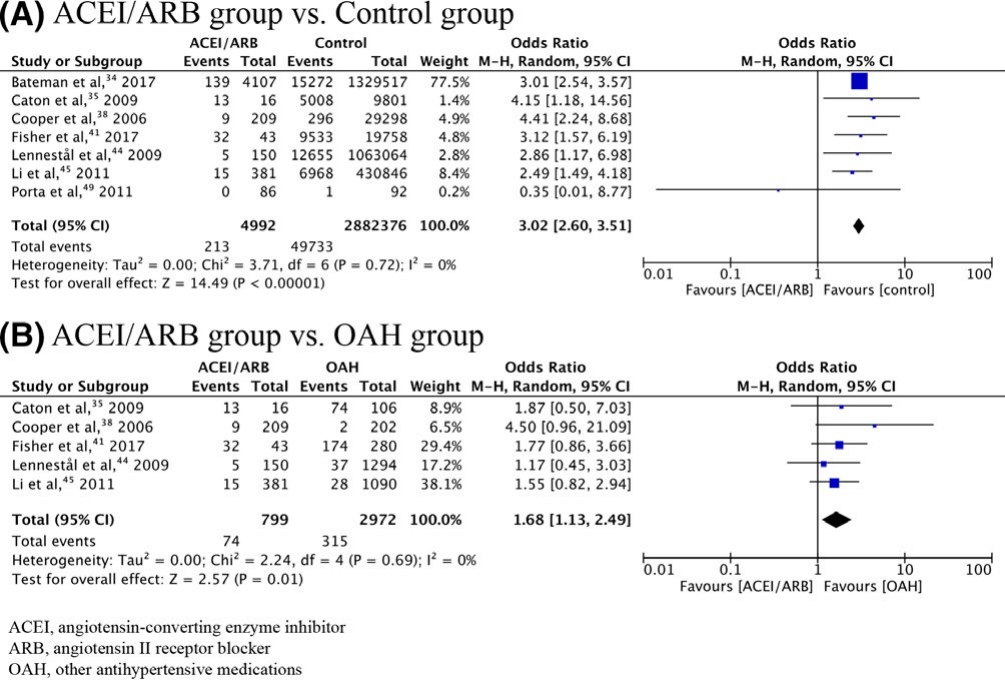
Forrest plot of CVS malformations in first trimester‐only exposure to ACEI/ARB compared with control and OAH

Meta‐analysis of 17 included studies found a significant association between overall congenital malformations and prenatal exposure to ACEIs/ARBs (OR = 2.16, 95% CI = 1.72‐2.71, *P* < .0001, calculated RR = 2.06; Table 2). A cumulative meta‐analysis demonstrated that the addition of subsequent studies had little effect on the OR, but simply narrowed the 95% CI (Figure S1). The significant relationship still existed when analysis was limited to studies with the first trimester‐only exposure (OR = 1.94, 95% CI = 1.71‐2.21, *P* < .0001, calculated RR = 1.91; Figure 2). The cumulative meta‐analysis displaying results accumulated over successive studies is shown in Figure S2. Cardiovascular system (CVS), central nervous system (CNS), and urogenital malformations were found to be associated with ACEI/ARB exposure during pregnancy (OR = 2.96, 95% CI = 2.57‐3.39, *P* < .0001, calculated RR = 2.87; OR = 2.02, 95% CI = 1.08‐3.78, *P* = .03, calculated RR = 2.01; OR = 4.57, 95% CI = 2.11‐9.89, *P* = .0001, calculated RR = 4.35, respectively). The significant association between ACEI/ARB exposure and CVS malformations was still present when analysis was limited to studies with the first trimester‐only exposure (OR = 3.02, 95% CI = 2.60‐3.51, *P* < .0001, calculated RR = 2.92; Figure 3).

**TABLE 2:** Adverse pregnancy outcomes following ACEI/ARB exposure compared with control

Outcomes	Studies included	Exposure		Heterogeneity		Effect measure		
		ACEIs/ARBs	Control	*χ* ^2^	*I* ^2^	OR	95% CI	*p* value
Exposure in any trimesters								
Congenital malformations								
Overall	17	538/6935	166295/3804799	0.0002	64%	2.16	(1.72, 2.71)	<.00001
CVS	9	244/5828	56389/3372581	0.71	0%	2.96	(2.57, 3.39)	<.0001
CNS	3	22/5014	5475/1800439	0.14	49%	2.02	(1.08, 3.78)	.03
Urogenital	2	7/141	1352/96903	0.81	0%	4.57	(2.11, 9.89)	.0001
LBW	3	101/639	27499/475076	0.001	85%	2.30	(1.20, 4.41)	.0004
Miscarriage	6	149/1180	254/3070	0.39	4%	1.63	(1.30, 2.05)	<.0001
ETOP	6	118/1180	145/3070	0.003	73%	2.54	(1.41, 4.59)	.02
Stillbirth	8	15/1474	24/4690	0.42	0%	2.36	(1.17, 4.76)	.02
Preterm delivery	9	321/1478	39071/478072	<0.00001	95%	1.69	(1.04, 2.76)	<.00001
Exposure in the first trimester only								
Congenital malformations								
Overall	14	400/6071	107994/3252689	0.41	4%	1.94	(1.71, 2.21)	<.00001
CVS	7	213/4992	49733/2882376	0.72	0%	3.02	(2.60, 3.51)	<.0001
CNS	3	16/4684	5250/1785430	0.08	61%	1.88	(0.73, 4.83)	.19
Urogenital	1	1/46	6/977	—	—	3.60	(0.42, 30.51)	.24
LBW	1	21/140	46/316	—	—	1.04	(0.59, 1.81)	.90
Miscarriage	6	149/1180	254/3070	0.39	4%	1.63	(1.30, 2.05)	<.0001
ETOP	6	118/1180	145/3070	0.003	73%	2.54	(1.41, 4.59)	.02
Stillbirth	8	15/1474	24/4690	0.42	0%	2.36	(1.17, 4.76)	.02
Preterm delivery	7	200/979	394/3312	0.0008	74%	1.26	(0.84, 1.91)	.26

Abbreviations: ACEIs, angiotensin‐converting enzyme inhibitors; ARBs, angiotensin II receptor blockers; CI, confidence interval; CNS, central nervous system; CVS, cardiovascular system; ETOP, elective termination of pregnancy; LBW, low birth weight; OR, odds ratio.

Other outcome measures that enabled analysis included LBW, miscarriage, ETOP, stillbirth, and preterm delivery, all of which were significantly associated with prenatal exposure to ACEIs/ARBs (Table 2). Miscarriage, ETOP, and stillbirth were also significantly related to ACEI/ARB exposure in the only first trimester of pregnancy (OR = 1.63, 95% CI = 1.30‐2.05, *P* < .0001, calculated RR = 1.55; OR = 2.54, 95% CI = 1.41‐4.59, *P* = .02, calculated RR = 2.37; OR = 2.36, 95% CI = 1.17‐4.76, *P* = .02, calculated RR = 2.34, respectively).

When comparing exposure to ACEIs/ARBs to nonexposure, the significant results were more or less similar to what was observed in the overall findings (Table S3). When comparing ACEI/ARB exposure to OAH exposure, the significant associations for most outcomes of interest were still existent when the analysis was limited to studies with the first trimester‐only exposure (Table S4).

Funnel plot asymmetries, indicative of the evidence of small‐study effects, were observed in the meta‐analyses of all the outcomes of interest, except for stillbirth (Figure S3). The formal tests suggested no significant asymmetry of the funnel plot for the effect estimate of overall congenital malformations (Rank correlation test, Kendall's Tau = −0.176, *P* = .349; Linear regression test, Z = −1.302, *P* = .193). When sensitivity analyses were applied, little changes on effect estimates were observed across all the outcomes of interest, indicative of robustness in the overall findings (Table S5). Prenatal exposure to ACEIs, but not ARBs, was found to be significantly associated with overall congenital malformations, LBW, miscarriage, ETOP, and preterm delivery.

## 4 DISCUSSION

To the best of our knowledge, this systematic review and meta‐analysis includes the largest dataset in the literature for the purpose of examining the associations between prenatal exposure to ACEIs/ARBs and adverse pregnancy outcomes, including both adverse maternal outcomes and neonatal birth defects. The first trimester‐only exposure to ACEIs/ARBs, previously presumably thought to be safe,^22^ was found to be significantly associated with adverse pregnancy outcomes, including overall and CVS congenital malformations. The overall results of this study may raise concerns about the potential dangers of ACEI/ARB use during early pregnancy.

The adverse pregnancy outcomes that occur following in utero exposure to ACEIs/ARBs may result either directly from the drugs or from underlying maternal illnesses. When the ACEI/ARB group was compared to the OAH group, the effect size was smaller than when it was compared to nonexposure. It is also possible that ACEIs/ARBs may be prescribed more often than other antihypertensive drug classes in hypertensive patients with diabetes because of their proven efficacy against the progression of diabetic nephropathy.^51^, ^52^ A hypertensive or diabetic disorder in pregnancy may itself be associated with adverse pregnancy outcomes without drug specificity and, thus, may act as a confounder in some observational studies included in our analysis.^53^, ^54^, ^55^ Moreover, patients with such underlying conditions tend to be older and may exhibit other comorbidities, including obesity, which may also be related to an elevated risk of adverse pregnancy outcomes.^56^, ^57^ Therefore, it should be kept in mind that there was a likelihood of the present meta‐analyses being confounded by some of these factors, for which some included studies might not adequately control.

Assumed the observed adverse pregnancy outcomes ascribed mainly to the drugs, the increased teratogenic risk could be conceivably attributed to inhibition of RAAS, a system that plays a key role in the embryogenic and fetal development of several organs/systems.^9^, ^58^, ^59^, ^60^ Not only does fetal RAAS blockade syndrome occur following ACEI/ARB exposure during the second and third trimesters of pregnancy it also may occur in those who are exposed to ACEIs/ARBs at the beginning of pregnancy.^15^, ^61^ Although there are unknown biologic mechanisms underlying adverse birth outcomes, inhibition of angiogenesis has been postulated to be a possible mechanism for the CVS malformations.^62^ Given limited knowledge on how ACEIs/ARBs might interfere with embryonic development during the critical period for organogenesis, further research is warranted to gain a better understanding of underlying mechanisms whereby the drugs might result in adverse pregnancy outcomes. Moreover, differential effects of ACEI/ARB exposure in the first trimester as compared to the second and third trimesters need further investigations.

Although it remains uncertain whether the elevated risk of adverse pregnancy outcomes observed in our analysis is specific to ACEIs/ARBs or related to maternal underlying conditions, this systematic review and meta‐analysis largely supports the current recommendations stating that women of reproductive age should be treated with ACEIs/ARBs only if absolutely indicated.^17^ Our findings may raise concerns about the potentially deleterious effects of prenatal exposure to ACEIs/ARBs during the first trimester of pregnancy. Given that numerous pregnancies are unplanned, there are formidable practical difficulties in avoiding first‐trimester ACEI/ARB exposure if the drugs are customarily used in female patients of reproductive age.^63^, ^64^ Clinical practitioners should treat those with the potential to become pregnant with the least teratogenic drug available.^25^, ^65^ Women of reproductive age whose condition is best treated with ACEIs/ARBs should be advised about the potential teratogenic risks of these drugs if they become pregnant. Effective contraception must be assured. However, if female patients inadvertently become pregnant while taking ACEIs/ARBs, clinical practitioners should instruct them to abruptly stop taking the drugs and offer alternatives.^66^ The only one randomized‐controlled trial included in our analysis suggested no significant association of adverse pregnancy outcomes with drug exposure when the patients discontinued an ARB within an estimated 8 weeks from the last menstrual period.^51^ It is reasonable to postulate that very short‐term cumulative exposure to ACEIs/ARBs during early pregnancy would be associated with better pregnancy outcomes; however, further investigation is required.

The results of this study should be interpreted with caution. First, asymmetric funnel plots, indicative of the evidence of small‐study effects (eg, publication bias), were observed in the meta‐analyses of most outcomes of interest. The formal tests for funnel plot asymmetry (either the Begg's rank correlation test or the Egger's linear regression test) are prone to type II errors (or false negative) in small meta‐analyses and, thus, the possibility of small‐study effects or publication bias cannot be ruled out.^67^ Although search terms being used were broad without being limited to specific study designs, it is conceivable that our analysis might have missed some pertinent studies which are, for example, only available in other databases (eg, Embase) or even be unpublished.^68^, ^69^ Positive studies reporting a teratogenic effect of the drugs may be more likely to be published than studies with null results.^70^ However, sensitivity analyses demonstrated no or little change on effect estimates, indicating the robustness of the results. Selective publications of studies may be of less concern to the validity of the present systematic review and meta‐analysis.^71^, ^72^ Second, it has been widely acknowledged in the literature that several observational studies on pregnancy outcome after drug exposure during early pregnancy often ignore left truncation and competing risks, leading to biased crude rates of miscarriage.^73^ Moreover, ETOP rates may reflect patients’ anxiety, including misunderstanding of drug risk, rather than the toxic effects of a drug. As a result, the meta‐analysis might misestimate the effects of prenatal exposure to ACEIs/ARBs, particularly when exposure is limited to the first trimester of pregnancy, on some outcomes of interest. Pharmacovigilance with regard to the exposure of newly pregnant women to their current medications will further provide more evidence on the association between ACEI/ARB use during the early stage of pregnancy and adverse pregnancy outcomes.

In conclusion, this comprehensive and quantitative analysis of the evidence available to date suggests an increased risk of adverse pregnancy outcomes, including congenital malformations, with prenatal exposure to ACEIs/ARBs, regardless of the trimester of pregnancy. Prescription of ACEIs/ARBs in women with the potential to become pregnant should be discouraged provided that there are alternative drugs with a more favorable risk/benefit profile to treat a condition. Large observational studies that are properly designed to adequately account for the role of confounders are necessary to confirm the results of this study. Further investigations are required to reveal possible pathogenic pathways leading to adverse pregnancy outcomes, particularly congenital birth defects, if confirmed, in those with first‐trimester exposure to ACEIs/ARBs.

## ETHICAL APPROVAL STATEMENT

This study is exempt from ethical review and received the certificate of exemption from the Research Ethics Committee of the Faculty of Medicine, Chiang Mai University.

## PATIENT CONSENT STATEMENT

Not applicable.

## CONFLICT OF INTEREST

The authors declare that they have no conflict of interest.

## AUTHORS' CONTRIBUTIONS

All authors contributed to the study conception and design. Data collection and analysis were performed by NB and NK. The first draft of the manuscript was written by NB and the manuscript was finalized by NK. All authors read and approved the final manuscript.

## Supporting information

Figure S1

Figure S2

Figure S3

Table S1

Table S2

Table S3

Table S4

Table S5

## Data Availability

All data used to support the findings of this study are available from the corresponding author upon reasonable request.
