# Supplementary figures and images for: Adverse pregnancy outcomes associated with first‐trimester exposure to angiotensin‐converting enzyme inhibitors or angiotensin II receptor blockers: A systematic review and meta‐analysis
Source: Pharmacol Res Perspect. 2020 Aug 19;8(5):e00644. doi: 10.1002/prp2.644 (PMC7438312; doi:10.1002/prp2.644)

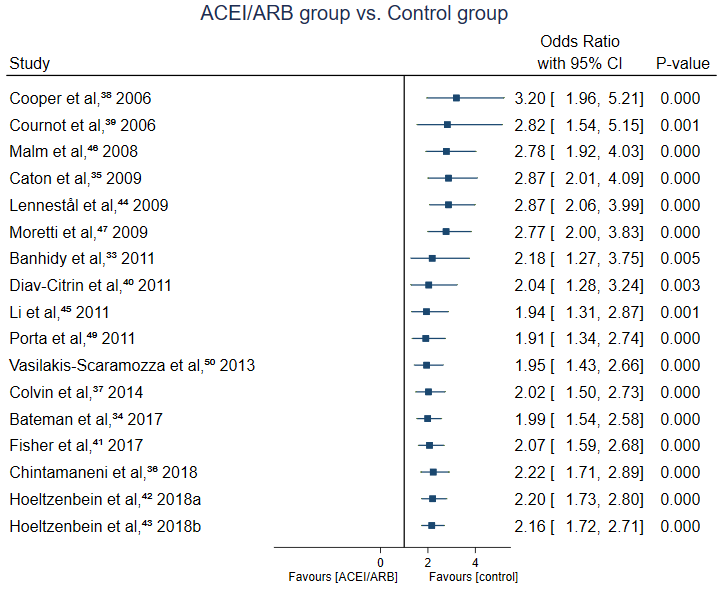

Supplement: Supplementary file 1 — Figure S1 [file PRP2-8-e00644-s001.tif]

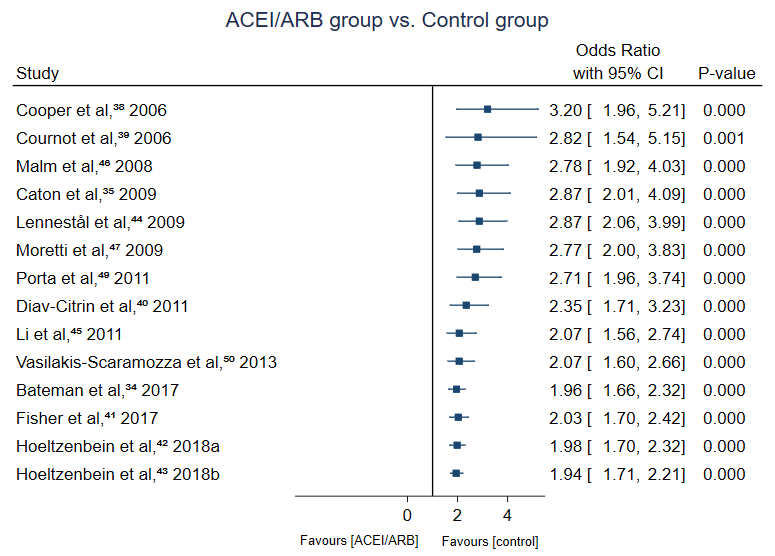

Supplement: Supplementary file 2 — Figure S2 [file PRP2-8-e00644-s002.tif]

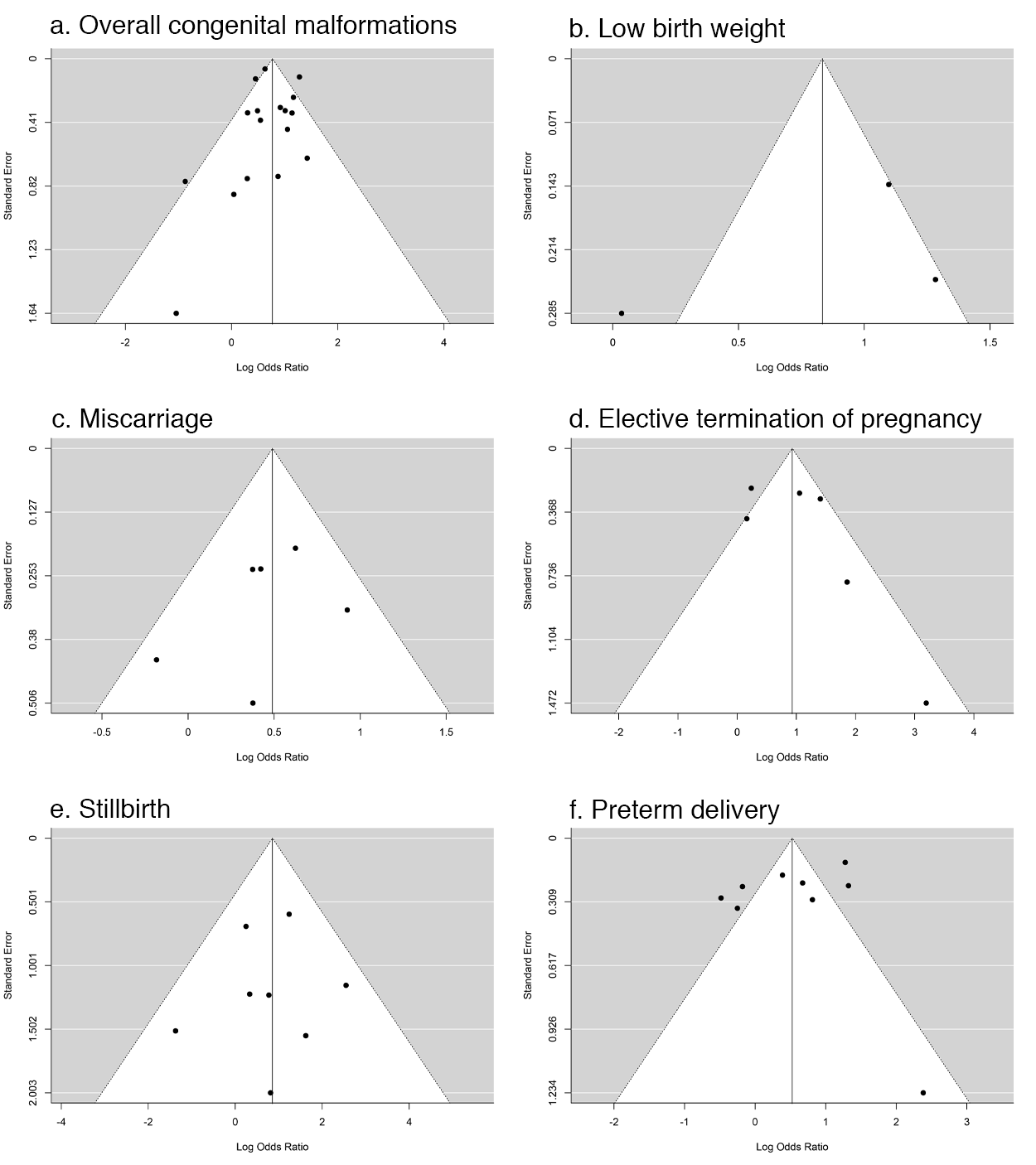

Supplement: Supplementary file 3 — Figure S3 [file PRP2-8-e00644-s003.tif]
