# Supplementary material for: Adverse pregnancy outcomes associated with first‐trimester exposure to angiotensin‐converting enzyme inhibitors or angiotensin II receptor blockers: A systematic review and meta‐analysis
Source: Pharmacol Res Perspect. 2020 Aug 19;8(5):e00644. doi: 10.1002/prp2.644 (PMC7438312; doi:10.1002/prp2.644)
Supplement: Supplementary file 4 — Table S1 [file PRP2-8-e00644-s004.docx]

**Table S1 Quality assessment by GRACE Checklist and Cochrane RoB 2**

| **Article** | **Domains** | | | | | | | | | | | | | | | | | | | | | |
| --- | --- | --- | --- | --- | --- | --- | --- | --- | --- | --- | --- | --- | --- | --- | --- | --- | --- | --- | --- | --- | --- | --- |
| **Observational study** | **D1** | | **D2** | | **D3** | | **D4** | | **D5** | | **D6** | | **M1** | | **M2** | | **M3** | | **M4** | | **M5** | |
| Ahmed et al,^32^ 2018 | - | | + | | + | | + | | + | | + | | - | | + | | + | | + | | - | |
| Banhidy et al,^33^ 2011 | - | | + | | + | | + | | + | | + | | - | | + | | + | | + | | - | |
| Bateman et al,^34^ 2017 | - | | + | | + | | + | | + | | + | | - | | + | | + | | + | | + | |
| Caton et al,^35^ 2009 | - | | + | | + | | + | | + | | + | | - | | + | | + | | + | | - | |
| Chintamaneni et al,^36^ 2018 | - | | + | | + | | + | | + | | + | | - | | + | | + | | + | | - | |
| Colvin et al,^37^ 2014 | - | | + | | + | | + | | + | | + | | - | | + | | + | | + | | - | |
| Cooper et al,^38^ 2006 | - | | + | | + | | + | | + | | + | | - | | + | | + | | + | | + | |
| Cournot et al,^39^ 2006 | - | | - | | + | | - | | + | | + | | - | | + | | - | | + | | - | |
| Diav-Citrin et al,^40^ 2011 | + | | + | | + | | + | | + | | + | | - | | + | | + | | + | | - | |
| Fisher et al,^41^ 2017 | - | | + | | + | | + | | + | | + | | - | | + | | + | | + | | - | |
| Hoeltzenbein et al,^42^ 2018a | + | | + | | + | | + | | + | | + | | - | | + | | + | | + | | + | |
| Hoeltzenbein et al,^43^ 2018b | + | | + | | + | | + | | + | | + | | - | | + | | + | | + | | + | |
| Lennestål et al,^44^ 2009 | - | | + | | + | | + | | + | | + | | - | | + | | + | | + | | - | |
| Li et al,^45^ 2011 | + | | + | | + | | + | | + | | + | | - | | + | | + | | + | | + | |
| Malm et al,^46^ 2008 | - | | + | | + | | + | | + | | + | | - | | + | | + | | + | | - | |
| Moretti et al,^47^ 2012 | - | | - | | + | | - | | + | | + | | - | | + | | + | | + | | - | |
| Piper et al,^48^ 1992 | - | | - | | + | | - | | - | | - | | - | | - | | - | | + | | - | |
| Vasilakis-Scaramozza et al,^50^ 2013 | - | | + | | + | | + | | + | | + | | - | | + | | + | | + | | - | |
| **Randomized-controlled study** | **Domains** | | | | | | | | | | | | | | | | | | | | | |
| Porta et al,^49^ 2011 | Bias arising from the randomization process | | | Bias due to deviations from intended interventions | | | | | | | Bias due to missing outcome data | | | | Bias in measurement of the outcome | | | | | Bias in selection of the reported result | | |
| Signaling question | 1.1 | 1.2 | 1.3 | 2.1 | 2.2 | 2.3 | 2.4 | 2.5 | 2.6 | 2.7 | 3.1 | 3.2 | 3.3 | 3.4 | 4.1 | 4.2 | 4.3 | 4.4 | 4.5 | 5.1 | 5.2 | 5.3 |
| Response options | Yes | Yes | No | No | No | - | - | - | Yes | - | Yes | - | - | - | No | No | No | - | - | Yes | No | No |
|  | Low | Low | Low | Low | Low | - | - | - | Low | - | Low | - | - | - | Low | Low | Low | - | - | Low | Low | Low |
| Risk of material bias | Low | | | Low | | | | | | | Low | | | | Low | | | | | Low | | |
| Risk of bias | Low | | | | | | | | | | | | | | | | | | | | | |
