# Supplementary material for: Adverse pregnancy outcomes associated with first‐trimester exposure to angiotensin‐converting enzyme inhibitors or angiotensin II receptor blockers: A systematic review and meta‐analysis
Source: Pharmacol Res Perspect. 2020 Aug 19;8(5):e00644. doi: 10.1002/prp2.644 (PMC7438312; doi:10.1002/prp2.644)
Supplement: Supplementary file 5 — Table S2 [file PRP2-8-e00644-s005.docx]

**Table S2 Summary of the results in included studies**

| **Study** | **Any congenital malformation** | **LBW** | **Miscarriage** | **ETOP** | **Stillbirth** | **Preterm delivery** |
| --- | --- | --- | --- | --- | --- | --- |
| Ahmed et al,^32^ 2018 |  | ↔ |  |  |  | ↔ |
| Banhidy et al,^33^ 2011 | ↔ |  |  |  |  |  |
| Bateman et al,^34^ 2017 | ↔ |  |  |  |  |  |
| Caton et al,^35^ 2009 | ↑ |  |  |  |  |  |
| Chintamaneni et al,^36^ 2018 | ↑ | ↑ |  |  |  | ↑ |
| Colvin et al,^37^ 2014 | ↑ |  |  |  |  |  |
| Cooper et al,^38^ 2006 | ↑ |  |  |  |  |  |
| Cournot et al,^39^ 2006 | ↔ |  |  |  |  |  |
| Diav-Citrin et al,^40^ 2011 | ↔ | ↑ | ↑ | ↑ | ↔ | ↑ |
| Fisher et al,^41^ 2017 | ↑ |  |  |  |  |  |
| Hoeltzenbein et al,^42^ 2018a | ↑ |  | ↔ | ↔ |  | ↑ |
| Hoeltzenbein et al,^43^ 2018b | ↔ |  | ↔ | ↑ |  | ↔ |
| Lennestål et al,^44^ 2009 | ↑ | ↑ |  |  |  | ↑ |
| Li et al,^45^ 2011 | ↔ |  |  |  |  |  |
| Malm et al,^46^ 2008 | ↑ |  |  |  |  |  |
| Moretti et al,^47^ 2012 | ↔ | ↔ | ↑ |  |  | ↑ |
| Piper et al,^48^ 1992 | ↑ |  |  |  |  | ↑ |
| Porta et al,^49^ 2011 | ↔ |  | ↔ | ↔ | ↔ | ↔ |
| Vasilakis-Scaramozza et al,^50^ 2013 | ↑ |  |  |  |  |  |

↔, no association with ACEI/ARB use; ↑, significant association with ACEI/ARB use.
