# Supplementary material for: Adverse pregnancy outcomes associated with first‐trimester exposure to angiotensin‐converting enzyme inhibitors or angiotensin II receptor blockers: A systematic review and meta‐analysis
Source: Pharmacol Res Perspect. 2020 Aug 19;8(5):e00644. doi: 10.1002/prp2.644 (PMC7438312; doi:10.1002/prp2.644)
Supplement: Supplementary file 6 — Table S3 [file PRP2-8-e00644-s006.docx]

**Table S3 Adverse pregnancy outcomes following ACEI/ARB exposure compared with non-exposure**

| **Outcomes** | **Studies included** | **Exposure** | | | **Heterogeneity** | | | **Effect measure** | | |
| --- | --- | --- | --- | --- | --- | --- | --- | --- | --- | --- |
|  |  | **ACEIs/ARBs** | **None** | **Chi^2^** | | ***I*^2^** | **OR** | | **95% CI** | ***p* value** |
| *Exposure in any trimesters* | | | | | | | | | | |
| Congenital malformations |  |  |  |  | |  |  | |  |  |
| Overall | 16 | 496/6443 | 171424/3782134 | 0.0005 | | 62% | 2.13 | | (1.67, 2.71) | <0.00001 |
| CVS | 9 | 244/5828 | 55413/3687994 | 0.77 | | 0% | 3.00 | | (2.61, 3.45) | <0.00001 |
| CNS | 3 | 22/5014 | 5241/1784157 | 0.15 | | 48% | 2.05 | | (1.10, 3.80) | 0.02 |
| Urogenital | 2 | 7/141 | 1350/96608 | 0.85 | | 0% | 4.61 | | (2.12, 10.00) | 0.0001 |
| LBW | 2 | 80/499 | 27453/474760 | 0.51 | | 0% | 3.14 | | (2.47, 3.99) | <0.00001 |
| Miscarriage | 6 | 149/1180 | 180/2180 | 0.59 | | 0% | 1.16 | | (1.27, 2.03) | <0.0001 |
| ETOP | 6 | 118/1180 | 105/2180 | 0.01 | | 65% | 2.46 | | (1.41, 4.29) | 0.001 |
| Stillbirth | 6 | 11/1180 | 7/2180 | 0.51 | | 0% | 2.27 | | (0.84, 6.15) | 0.11 |
| Preterm delivery | 7 | 265/1176 | 38808/476657 | <0.0001 | | 79% | 2.94 | | (1.89, 4.58) | <0.00001 |
| *Exposure in the first trimester only* | | | | | | | | | | |
| Congenital malformations |  |  |  |  | |  |  | |  |  |
| Overall | 13 | 389/5934 | 95824/2899709 | 0.42 | | 2% | 1.93 | | (1.70, 2.19) | <0.00001 |
| CVS | 7 | 213/4992 | 49418/2879404 | 0.71 | | 0% | 3.02 | | (2.60, 3.51) | <0.00001 |
| CNS | 3 | 16/4684 | 5241/1784157 | 0.08 | | 61% | 1.87 | | (0.73, 4.79) | 0.19 |
| Urogenital | 1 | 1/46 | 4/682 | - | | - | 3.77 | | (0.41, 34.40) | 0.24 |
| LBW | 0 | - | - | - | | - | - | | - | - |
| Miscarriage | 6 | 149/1180 | 180/2180 | 0.59 | | 0% | 1.16 | | (1.27, 2.03) | <0.0001 |
| ETOP | 6 | 118/1180 | 105/2180 | 0.01 | | 65% | 2.46 | | (1.41, 4.29) | 0.001 |
| Stillbirth | 6 | 11/1180 | 7/2180 | 0.51 | | 0% | 2.27 | | (0.84, 6.15) | 0.11 |
| Preterm delivery | 5 | 144/677 | 131/1897 | 0.0003 | | 81% | 2.81 | | (1.32, 5.99) | 0.007 |
