# Supplementary material for: Adverse pregnancy outcomes associated with first‐trimester exposure to angiotensin‐converting enzyme inhibitors or angiotensin II receptor blockers: A systematic review and meta‐analysis
Source: Pharmacol Res Perspect. 2020 Aug 19;8(5):e00644. doi: 10.1002/prp2.644 (PMC7438312; doi:10.1002/prp2.644)
Supplement: Supplementary file 7 — Table S4 [file PRP2-8-e00644-s007.docx]

**Table S4 Adverse pregnancy outcomes following ACEI/ARB exposure compared with exposure to other antihypertensive drugs**

| **Outcomes** | **Studies included** | **Exposure** | | **Heterogeneity** | | **Effect measure** | | |
| --- | --- | --- | --- | --- | --- | --- | --- | --- |
|  |  | **ACEIs/ARBs** | **OAH** | **Chi^2^** | ***I*^2^** | **OR** | **95% CI** | ***p* value** |
| *Exposure in any trimesters* | | | | | | | | |
| Congenital malformations |  |  |  |  |  |  |  |  |
| Overall | 11 | 139/1567 | 3830/25379 | 0.20 | 25% | 1.33 | (0.96, 1.84) | 0.09 |
| CVS | 5 | 87/1136 | 976/18417 | 0.20 | 33% | 1.36 | (0.87, 2.14) | 0.18 |
| CNS | 2 | 11/907 | 234/16282 | 0.16 | 49% | 1.42 | (0.21, 9.59) | 0.72 |
| Urogenital | 1 | 1/46 | 2/295 | - | - | 3.26 | (0.29, 36.64) | 0.34 |
| LBW | 1 | 21/140 | 46/316 | - | - | 1.04 | (0.59, 1.81) | 0.90 |
| Miscarriage | 4 | 124/901 | 74/890 | 0.04 | 64% | 1.85 | (1.07, 3.20) | 0.03 |
| ETOP | 4 | 83/901 | 21/890 | 0.01 | 73% | 6.63 | (1.74, 25.39) | 0.006 |
| Stillbirth | 6 | 11/1195 | 18/2510 | 0.19 | 35% | 1.49 | (0.51, 4.41) | 0.47 |
| Preterm delivery | 6 | 175/874 | 263/1415 | 0.50 | 0% | 0.85 | (0.68, 1.06) | 0.14 |
| *Exposure in the first trimester only* | | | | | | | | |
| Congenital malformations |  |  |  |  |  |  |  |  |
| Overall | 10 | 137/1557 | 403/4128 | 0.69 | 0% | 1.48 | (1.13, 1.92) | 0.004 |
| CVS | 5 | 74/799 | 315/2972 | 0.69 | 0% | 1.68 | (1.13, 2.49) | 0.01 |
| CNS | 2 | 5/577 | 9/1273 | 0.16 | 50% | 1.49 | (0.16, 14.14) | 0.73 |
| Urogenital | 1 | 1/46 | 2/295 | - | - | 3.26 | (0.29, 36.64) | 0.34 |
| LBW | 1 | 21/140 | 46/316 | - | - | 1.04 | (0.59, 1.81) | 0.90 |
| Miscarriage | 4 | 124/901 | 74/890 | 0.04 | 64% | 1.85 | (1.07, 3.20) | 0.03 |
| ETOP | 4 | 83/901 | 21/890 | 0.01 | 73% | 6.63 | (1.74, 25.39) | 0.006 |
| Stillbirth | 6 | 11/1195 | 18/2510 | 0.19 | 35% | 1.49 | (0.51, 4.41) | 0.47 |
| Preterm delivery | 6 | 175/874 | 263/1415 | 0.50 | 0% | 0.85 | (0.68, 1.06) | 0.14 |
