# Supplementary material for: Adverse pregnancy outcomes associated with first‐trimester exposure to angiotensin‐converting enzyme inhibitors or angiotensin II receptor blockers: A systematic review and meta‐analysis
Source: Pharmacol Res Perspect. 2020 Aug 19;8(5):e00644. doi: 10.1002/prp2.644 (PMC7438312; doi:10.1002/prp2.644)
Supplement: Supplementary file 8 — Table S5 [file PRP2-8-e00644-s008.docx]

**Table S5 Subgroup and sensitivity analyses**

| **Outcome** | **Condition for analysis** | **Studies included** | **Fixed-effect meta-analysis** | | | | **Random-effects meta-analysis** | | |
| --- | --- | --- | --- | --- | --- | --- | --- | --- | --- |
|  |  |  | **OR** | **95% CI** | ***p* value** | **OR** | | **95% CI** | ***p* value** |
| Overall congenital malformations | All | 17 | 2.05 | [1.87, 2.25] | <0.00001 | 2.16 | | [1.72, 2.71] | <0.00001 |
|  | *Study design* | | | | | | | | |
|  | Prospective cohort studies | 5 | 1.50 | [1.02, 2.19] | 0.04 | 1.50 | | [1.03, 2.20] | 0.04 |
|  | Retrospective cohort studies | 8 | 2.09 | [1.90, 2.31] | <0.00001 | 2.44 | | [1.83, 3.25] | <0.00001 |
|  | Case-controlled studies | 3 | 2.46 | [1.46, 4.12] | 0.0007 | 2.01 | | [0.64, 6.36] | 0.23 |
|  | Randomized-controlled trials | 1 | 0.35 | [0.01, 8.77] | 0.52 | 0.35 | | [0.01, 8.77] | 0.52 |
|  | *Drug classes* | | | | | | | | |
|  | Only ACEIs | 13 | 2.03 | [1.85, 2.24] | <0.00001 | 2.12 | | [1.64, 2.74] | <0.00001 |
|  | Only ARBs | 4 | 1.75 | [0.98, 3.14] | 0.06 | 1.63 | | [0.88, 3.01] | 0.12 |
|  | *Exclusion of a single study* | | | | | | | | |
|  | Banhidy et al,^33^ 2011 | 16 | 2.07 | [1.89, 2.28] | <0.00001 | 2.23 | | [1.79, 2.78] | <0.00001 |
|  | Bateman et al,^34^ 2017 | 16 | 2.26 | [1.97, 2.58] | <0.00001 | 2.18 | | [1.65, 2.86] | <0.00001 |
|  | Caton et al,^35^ 2009 | 16 | 2.04 | [1.86, 2.24] | <0.00001 | 2.12 | | [1.68, 2.67] | <0.00001 |
|  | Chintamaneni et al,^36^ 2018 | 16 | 1.88 | [1.69, 2.08] | <0.00001 | 1.97 | | [1.66, 2.34] | <0.00001 |
|  | Colvin et al,^37^ 2014 | 16 | 2.04 | [1.86, 2.24] | <0.00001 | 2.12 | | [1.67, 2.69] | <0.00001 |
|  | Cooper et al,^38^ 2006 | 16 | 2.03 | [1.84, 2.23] | <0.00001 | 2.08 | | [1.64, 2.64] | <0.00001 |
|  | Cournot et al,^39^ 2006 | 16 | 2.06 | [1.87, 2.26] | <0.00001 | 2.18 | | [1.73, 2.75] | <0.00001 |
|  | Diav-Citrin et al,^40^ 2011 | 16 | 2.07 | [1.89, 2.28] | <0.00001 | 2.23 | | [1.76, 2.82] | <0.00001 |
|  | Fisher et al,^41^ 2017 | 16 | 2.03 | [1.85, 2.23] | <0.00001 | 2.10 | | [1.66, 2.67] | <0.00001 |
|  | Hoeltzenbein et al,^42^ 2018a | 16 | 2.06 | [1.88, 2.26] | <0.00001 | 2.18 | | [1.72, 2.77] | <0.00001 |
|  | Hoeltzenbein et al,^43^ 2018b | 16 | 2.06 | [1.88, 2.27] | <0.00001 | 2.20 | | [1.73, 2.79] | <0.00001 |
|  | Lennestal et al,^44^ 2009 | 16 | 2.05 | [1.86, 2.25] | <0.00001 | 2.13 | | [1.68, 2.69] | <0.00001 |
|  | Li et al,^45^ 2011 | 16 | 2.15 | [1.94, 2.38] | <0.00001 | 2.25 | | [1.75, 2.88] | <0.00001 |
|  | Malm et al,^46^ 2008 | 16 | 2.04 | [1.86, 2.25] | <0.00001 | 2.13 | | [1.67, 2.71] | <0.00001 |
|  | Moretti et al,^47^ 2009 | 16 | 2.06 | [1.88, 2.26] | <0.00001 | 2.18 | | [1.73, 2.75] | <0.00001 |
|  | Porta et al,^49^ 2011 | 16 | 2.06 | [1.88, 2.26] | <0.00001 | 2.18 | | [1.73, 2.74] | <0.00001 |
|  | Vasilakis-Scaramozza et al,^50^ 2013 | 16 | 2.05 | [1.87, 2.25] | <0.00001 | 2.15 | | [1.70, 2.72] | <0.00001 |
| LBW | All | 3 | 2.43 | [1.94, 3.06] | <0.00001 | 2.30 | | [1.20, 4.41] | 0.01 |
|  | *Study design* | | | | | | | | |
|  | Prospective cohort studies | - | - | - | - | - | | - | - |
|  | Retrospective cohort studies | 3 | 2.43 | [1.94, 3.06] | <0.00001 | 2.30 | | [1.20, 4.41] | 0.01 |
|  | Case-controlled studies | - | - | - | - | - | | - | - |
|  | Randomized-controlled trials | - | - | - | - | - | | - | - |
|  | *Drug classes* | | | | | | | | |
|  | Only ACEIs | 3 | 2.60 | [2.60, 3.28] | <0.00001 | 2.26 | | [1.16, 4.39] | 0.02 |
|  | Only ARBs | 1 | 1.27 | [0.65, 2.50] | 0.49 | 1.27 | | [0.65, 2.50] | 0.49 |
|  | *Exclusion of a single study* | | | | | | | | |
|  | Ahmed et al,^32^ 2018 | 2 | 3.13 | [2.46, 3.98] | <0.00001 | 3.14 | | [2.47, 3.99] | <0.00001 |
|  | Chintamaneni et al,^36^ 2018 | 2 | 1.83 | [1.25, 2.68] | 0.002 | 1.95 | | [0.56, 6.83] | 0.30 |
|  | Colvin et al,^37^ 2014 | 2 | 2.23 | [1.72, 2.88] | <0.00001 | 1.81 | | [0.63, 5.24] | 0.27 |
| Miscarriage | All | 6 | 1.63 | [1.31, 2.03] | <0.0001 | 1.63 | | [1.30, 2.05] | <0.0001 |
|  | *Study design* | | | | | | | | |
|  | Prospective cohort studies | 5 | 1.73 | [1.38, 2.16] | <0.00001 | 1.72 | | [1.37, 2.17] | <0.00001 |
|  | Retrospective cohort studies | - | - | - | - | - | | - | - |
|  | Case-controlled studies | - | - | - | - | - | | - | - |
|  | Randomized-controlled trials | 1 | 0.83 | [0.37, 1.90] | 0.66 | 0.83 | | [0.37, 1.90] | 0.66 |
|  | *Drug classes* | | | | | | | | |
|  | Only ACEIs | 3 | 1.49 | [1.08, 2.04] | 0.01 | 1.49 | | [1.08, 2.04] | 0.01 |
|  | Only ARBs | 2 | 1.59 | [1.12, 2.26] | 0.01 | 1.36 | | [0.63, 2.94] | 0.44 |
|  | *Exclusion of a single study* | | | | | | | | |
|  | Cournot et al,^39^ 2006 | 5 | 1.64 | [1.31, 2.05] | <0.0001 | 1.63 | | [1.26, 2.12] | 0.0003 |
|  | Diav-Citrin et al,^40^ 2011 | 5 | 1.66 | [1.30, 2.13] | <0.0001 | 1.65 | | [1.22, 2.21] | 0.001 |
|  | Hoeltzenbein et al,^42^ 2018a | 5 | 1.68 | [1.31, 2.15] | <0.0001 | 1.67 | | [1.25, 2.23] | 0.0005 |
|  | Hoeltzenbein et al,^43^ 2018b | 5 | 1.53 | [1.18, 2.00] | 0.002 | 1.53 | | [1.15, 2.05] | 0.004 |
|  | Moretti et al,^47^ 2009 | 5 | 1.53 | [1.21, 1.94] | 0.0004 | 1.54 | | [1.22, 1.95] | 0.0003 |
|  | Porta et al,^49^ 2011 | 5 | 1.73 | [1.38, 2.16] | <0.0001 | 1.72 | | [1.37, 2.17] | <0.0001 |
| ETOP | All | 6 | 2.22 | [1.72, 2.88] | <0.0001 | 2.54 | | [1.41, 4.59] | 0.002 |
|  | *Study design* | | | | | | | | |
|  | Prospective cohort studies | 5 | 2.42 | [1.84, 3.18] | <0.00001 | 3.04 | | [1.56, 5.94] | 0.001 |
|  | Retrospective cohort studies | - | - | - | - | - | | - | - |
|  | Case-controlled studies | - | - | - | - | - | | - | - |
|  | Randomized-controlled trials | 1 | 1.18 | [0.53, 2.61] | 0.69 | 1.18 | | [0.53, 2.61] | 0.69 |
|  | *Drug classes* | | | | | | | | |
|  | Only ACEIs | 3 | 3.54 | [2.45, 5.11] | <0.00001 | 3.48 | | [2.41, 5.03] | <0.00001 |
|  | Only ARBs | 2 | 1.25 | [0.84, 1.84] | 0.27 | 1.25 | | [0.84, 1.85] | 0.27 |
|  | *Exclusion of a single study* | | | | | | | | |
|  | Cournot et al,^39^ 2006 | 5 | 2.11 | [1.62, 2.75] | <0.00001 | 2.29 | | [1.24, 4.25] | 0.008 |
|  | Diav-Citrin et al,^40^ 2011 | 5 | 2.04 | [1.51, 2.76] | <0.00001 | 2.58 | | [1.18, 5.66] | 0.02 |
|  | Hoeltzenbein et al,^42^ 2018a | 5 | 1.94 | [1.45, 2.60] | <0.00001 | 2.21 | | [1.15, 4.25] | 0.02 |
|  | Hoeltzenbein et al,^43^ 2018b | 5 | 3.03 | [2.18, 4.21] | <0.00001 | 3.07 | | [1.68, 5.61] | 0.0003 |
|  | Moretti et al,^47^ 2009 | 5 | 2.11 | [1.63, 2.75] | <0.00001 | 2.32 | | [1.30, 4.13] | 0.004 |
|  | Porta et al,^49^ 2011 | 5 | 2.42 | [1.84, 3.18] | <0.00001 | 3.04 | | [1.56, 5.94] | 0.001 |
| Stillbirth | All | 8 | 2.11 | [1.10, 4.08] | 0.03 | 2.36 | | [1.17, 4.76] | 0.02 |
|  | *Study design* | | | | | | | | |
|  | Prospective cohort studies | 5 | 1.76 | [0.77, 4.00] | 0.18 | 1.96 | | [0.62, 6.17] | 0.25 |
|  | Retrospective cohort studies | 2 | 3.45 | [1.07, 11.14] | 0.04 | 3.45 | | [1.07, 11.14] | 0.04 |
|  | Case-controlled studies | - | - | - | - | - | | - | - |
|  | Randomized-controlled trials | 1 | 2.17 | [0.19, 24.33] | 0.53 | 2.17 | | [0.19, 24.33] | 0.53 |
|  | *Drug classes* | | | | | | | | |
|  | Only ACEIs | 5 | 2.39 | [1.06, 5.40] | 0.04 | 2.35 | | [0.91, 6.07] | 0.08 |
|  | Only ARBs | 3 | 1.74 | [0.56, 5.40] | 0.34 | 1.73 | | [0.55, 5.39] | 0.35 |
|  | *Exclusion of a single study* | | | | | | | | |
|  | Ahmed et al,^32^ 2018 | 7 | 2.11 | [1.10, 4.08] | 0.03 | 2.36 | | [1.17, 4.76] | 0.02 |
|  | Cournot et al,^39^ 2006 | 7 | 1.97 | [1.00, 3.90] | 0.05 | 2.22 | | [0.99, 4.97] | 0.05 |
|  | Diav-Citrin et al,^40^ 2011 | 7 | 2.50 | [1.17, 5.36] | 0.02 | 2.94 | | [1.30, 6.68] | 0.01 |
|  | Hoeltzenbein et al,^42^ 2018a | 7 | 1.73 | [0.85, 3.52] | 0.13 | 1.97 | | [0.94, 4.12] | 0.07 |
|  | Hoeltzenbein et al,^43^ 2018b | 7 | 2.19 | [1.10, 4.34] | 0.02 | 2.47 | | [1.08, 5.65] | 0.03 |
|  | Lennestal et al,^44^ 2009 | 7 | 1.80 | [0.83, 3.92] | 0.14 | 1.93 | | [0.77, 4.82] | 0.16 |
|  | Moretti et al,^47^ 2009 | 7 | 2.67 | [1.33, 5.35] | 0.006 | 2.69 | | [1.31, 5.54] | 0.007 |
|  | Porta et al,^49^ 2011 | 7 | 2.11 | [1.07, 4.17] | 0.03 | 2.35 | | [1.00, 5.48] | 0.05 |
| Preterm delivery | All | 9 | 1.84 | [1.60, 2.13] | <0.00001 | 1.69 | | [1.04, 2.76] | 0.03 |
|  | *Study design* | | | | | | | | |
|  | Prospective cohort studies | 5 | 1.46 | [1.18, 1.82] | 0.0006 | 1.50 | | [1.01, 2.24] | 0.04 |
|  | Retrospective cohort studies | 3 | 2.59 | [2.13, 3.16] | <0.00001 | 2.45 | | [0.98, 6.14] | <0.00001 |
|  | Case-controlled studies | - | - | - | - | - | | - | - |
|  | Randomized-controlled trials | 1 | 0.78 | [0.40, 1.51] | 0.46 | 0.78 | | [0.40, 1.51] | 0.46 |
|  | *Drug classes* | | | | | | | | |
|  | Only ACEIs | 6 | 2.18 | [1.83, 2.59] | <0.00001 | 1.92 | | [0.99, 3.70] | 0.05 |
|  | Only ARBs | 3 | 1.14 | [0.86, 1.51] | 0.38 | 0.98 | | [0.57, 1.69] | 0.95 |
|  | *Exclusion of a single study* | | | | | | | | |
|  | Ahmed et al,^32^ 2018 | 8 | 2.03 | [1.75, 2.36] | <0.00001 | 1.92 | | [1.20, 3.08] | 0.006 |
|  | Chintamaneni et al,^36^ 2018 | 8 | 1.41 | [1.18, 1.68] | 0.0002 | 1.50 | | [0.94, 2.39] | 0.09 |
|  | Colvin et al,^37^ 2014 | 8 | 1.74 | [1.50, 2.02] | <0.00001 | 1.52 | | [0.89, 2.58] | 0.12 |
|  | Diav-Citrin et al,^40^ 2011 | 8 | 1.83 | [1.57, 2.13] | <0.00001 | 1.67 | | [0.95, 2.93] | 0.08 |
|  | Hoeltzenbein et al,^42^ 2018a | 8 | 2.04 | [1.75, 2.38] | <0.00001 | 1.87 | | [1.14, 3.05] | 0.01 |
|  | Hoeltzenbein et al,^43^ 2018b | 8 | 1.94 | [1.65, 2.27] | <0.00001 | 1.74 | | [0.99, 3.06] | 0.06 |
|  | Moretti et al,^47^ 2009 | 8 | 1.82 | [1.57, 2.11] | <0.00001 | 1.64 | | [0.95, 2.81] | 0.07 |
|  | Porta et al,^49^ 2011 | 8 | 1.94 | [1.67, 2.24] | <0.00001 | 1.86 | | [1.12, 3.08] | 0.02 |
|  | Vasilakis-Scaramozza et al,^50^ 2013 | 8 | 1.84 | [1.59, 2.12] | <0.00001 | 1.60 | | [0.97, 2.61] | 0.06 |
